# The Foreign Journals

**Published:** 1836-04

**Authors:** 


### Art. XX.—THE FOREIGN JOURNALS. No. II.

### GERMAN JOURNALS.

7.—*Magazin für die gesammte Heilkunde. Herausgegeben* von J. N. Rust.—*Berlin,* 1836.
*Rust's Magazine of Medical Science.*

The name of the Editor of this magazine, and of the magazine itself, must be familiar to all who have attended to medical literature during the present century. If not the oldest of the existing journals, it is among the oldest, and is one of the best known and most worthy of being known. It is, like most of the German journals, of a comparatively small size, being small 8vo., and each number containing from ten to twelve sheets, or from 160 to 190 pages. It appears at no fixed periods. Three numbers constitute a volume, and cost three dollars. The number now before us, the first of 1836, commences vol. xlvi., and consequently is the 138th, reckoning from the beginning of the series.

This journal is composed chiefly of original memoirs in all the departments of medicine, and contains also a small department under the title of *Miscellanies,* which gives brief articles of medical intelligence and other notices of temporary interest. Among these we observe frequent official documents of government referring to medical affairs. On the wrapper of this journal is the following notice, of which we will only remark that it refers to a practice common in Germany, and which tends to account for the general comparative goodness of the original communications found in the periodical publications of Germany. If it were adopted by the journals in this country, we should find their pages less occupied by accounts of single and isolated cases, which are so frequently of little or no value. "Communications to be addressed, &c. Every paper judged proper to be admitted will be inserted as soon as possible, and an adequate *honorarium* therefor, be paid immediately after the completion of the volume, either in cash or by order, according to circumstances, or the wish of the party. The copyright of the article, after the expiration of one year, belongs to the author." The great value of many of the original communications in this Journal would seem to indicate that these *honoraries* are effective incitements to even the most distinguished men to communicate, through this medium, the results of their observation and experience. Why should not Original Communications be paid for in England, as well as Critical Articles?

8.—*Neue Zeitschrift für Geburtskunde. Herausgegeben* von Busch, D'Outrepont, *und* Ritgen.—*Berlin,* 1836.
*New Journal of Midwifery.* Edited by Von Busch, &c.

The duration of this Journal has not as yet belied its title of new, as it has only reached its ninth Number. It is, however, only a new series of a Journal of the same name, with the exception of the initial word, which commenced ten years since, and had reached its seventh volume, when it was superseded by the present publication; D'Outrepont having succeeded Mende in the co-editorship. It is of the usual 8vo. form, contains 160 pages in each number, of which three constitute a volume, of the price of three dollars. It does not appear oftener than four times in the year. It is exclusively, or almost exclusively, devoted to obstetrical subjects, and contains many papers of great value.

A small portion of each number, under the head "Literatur," is set apart for notices of books; but these are for the most part scanty and unsatisfactory. The Journal, however, is a very valuable one.

9.—*Zeitschrift für die gesammte Medicin mit besonderer rücksicht auf hospitalpraxis und ausländische Literatur. Herausgegeben* von J. F. DIEFFENBACH, J. C. G. FRICKE, *und* F. W. OPPENHEIM. —*Hamburg*, 1836. 8vo. Heft I. II., pp. 136, 152.

*Journal of Medicine in general, with particular reference to Hospital Practice and Foreign Literature.* Edited by, &c.

This is quite a new journal, having commenced only with the present year. It is, however, to be regarded as a continuation of the well known *Magazin der Ausländischen Literatur der Gesammten heilkunde*, so long edited at Hamburg by Drs. Gerson and Julius, only under a new title and management, and with some modification of the original plan. It is published monthly, and is neatly got up, with good paper and a clear Roman type. Like its predecessor, it is chiefly devoted to the task of making known FOREIGN MEDICINE to the profession in Germany, by means of *Reviews* and *Bibliographical Notices* of foreign books, *Selections from Foreign Journals*, and *Foreign Correspondence;* but it also contains *Original Memoirs.* The plan of this journal is excellent, and, judging from the execution of the first two parts now before us, and from the learning, talents, and professional eminence of the distinguished Editors, we venture to prophecy that it will be one of the most valuable and successful journals published in Germany. We recommend such of our book-writing friends in this country as are desirous of seeing the judgment of enlightened foreigners on their productions, to take in this journal, and also to forward to their agents in this country such works as they are desirous of having noticed by them. Owing to the comparatively small proportion of original memoirs, we shall derive from it fewer materials for our Third Department than from other journals of really inferior merit.

### ITALIAN JOURNALS.

4.—*L'Eclettico di Adone Palmierj, Giornale di Medicina, Chirurgia, Economia Domestica, Arte Industriale, Agricoltura, &c.*—*Roma.* Fol.

*The Eclectic of Adone Palmieri, Journal of Medicine, &c.*

This is a new journal, the first number having appeared only in September last. It is a weekly publication, of four small folio pages and large print, and probably contains altogether as much as two pages of the *Lancet.* It is published every Saturday, and costs fifteen *paoli* per annum. It is edited by the Professor (Palmieri) whose name it bears. This is truly a most jejune publication, made up chiefly of trifling extracts, or original cases more trifling still. Such a work could not live a month in any of the northern countries, and we hope, for the honour of Italy, it cannot long survive in Rome. We are tempted to translate a portion of a case entitled "A most severe disease happily cured," from No. 9, 31 Oct., as a specimen of the strange bombast in which the Italians permit themselves to indulge, even in such sober matters as those of physic; and our translation shall be as literal as possible, in order to preserve something of the smack of the original.

"Case. 'It is better that I kill myself with this sharp poignard, since intolerable are become the dire palpitations of my oppressed heart!' Such were the desperate expressions which the groaning Count di —— was pouring forth with hoarse voice, as I unexpectedly entered his chamber. His countenance was shrunk and discomposed, his look was wild, his bosom bare, his hair dishevelled, his beard unshorn. He reclined beside a table, the *left* elbow resting on it, and the hand of the same side supporting his head. Without appetite, he passed his nights unsleeping, his bowels burning and his brain boiling; and by day he busied himself with various unconnected matters, in a sort of fury. He was hastening to the tomb; talked only of lugubrious objects, of horrid spectres, and disgusted with his bitter fate, he expired from his lungs *billions* (sic) of sighs! It was, nevertheless, to no purpose that I searched for morbid symptoms to establish the diagnosis of some physical malady. He was attacked with that disease, which can render haggard the lovely virgin, with that disease which was the torment of Petrarch and Tasso, and to which fall equal victims philosophers and fools, shepherds and kings," &c.

In a word, the poor count was in love! and the marvellous cure effected by this grandiloquent leech consisted in sending him to the country to travel, work, hunt, &c.

We trust to see no more of *L'Eclettico di Adone Palmierj.*

5.—*Bullettino delle Scienze Mediche, pubblicato per cura della Società Medico-Chirurgica di Bologna, e redatto dai Socj* Prof. Baroni, Dott. Breventani, &c.—*Bologna.* 8vo.

*Bulletin of the Medical Sciences, published under the superintendence of the Medico-Chirurgical Society of Bologna, and edited by, &c.*

This journal is published monthly at Bologna, and contains on an average four sheets or sixty-four pages, in 8vo. Six numbers form a volume, and the price of the twelve annual numbers is two Roman *Scudi*, or 10¾ Italian *Lire.* It has existed seven years, and completed its twelfth volume last December. This is, on the whole, a respectable journal, although, like most Italian journals, its original papers are greatly exceeded, both in number and value, by those selected from foreign journals. By far the most interesting portion of its contents are the Reports of the *Accademia delle Scienze,* and of the *Societa Medico-Chirurgica* of Bologna, which it gives regularly: and this is the department from which we are most likely to draw, in supplying materials for our Third Department.

### FRENCH JOURNALS.

6.—*Journal Hebdomadaire des Progrès des Sciences et Institutions Médicales.* Rédigé par une Société de Médecins et de Chirurgiens des Hôpitaux.

This, as its name implies, is one of the weekly journals of Paris, of the same size and something of the same appearance as our own *Medical Gazette.* It consists of two sheets, or thirty-two pages, 8vo., appears every Saturday, and the fifty-two annual numbers make four volumes, price twenty francs. It consists of four departments: 1, Original Memoirs; 2, Reviews; 3, Reports of the Medical Societies; 4, Miscellanies. With the last number of each month is given a very useful list of all the medical works published in France during that month, and which, under the name of *Le Bulletin Bibliographique,* is also sold separately, at three francs for the twelve monthly numbers. This journal commenced in October 1828, and is now consequently in its eighth year.

It was lately edited by MM. Bouillaud, Forget, and Vidal, but their names are now removed from the title-page.

The Journal Hebdomadaire often contains valuable papers, and combines, in a greater degree than some of the other weekly journals, subjects of more permanent interest with the lighter topics of the day. As is the case with other journals, its medical news are occasionally rather stale. For instance, in one of the numbers now before us, bearing date 14 November, 1835, we find, under the head *Variétés,* a lively description of the disturbance which took place at Sheffield, two years ago at least, and to which the readers of the *Journal* are attracted, as to an event of recent occurrence, by the title "Emeute et Destruction de l'Ecole de Médecine de Sheffield." We may also notice in this place, as an instance of it has just caught our eye, the extreme inaccuracy of our Parisian brethren in giving the names of foreigners, in their published writings. This must arise from mere carelessness and inattention, as they have the printed works before them to correct any sins of orthography which might flow in the first instance from the national mode of pronunciation.

### AMERICAN JOURNALS.

3.—*The United States Medical and Surgical Journal, conducted by a number of respectable Physicians in various parts of the United States.—New York.* 8vo.

This is a new journal, having only existed about two years. It is published monthly. Its general form and style of getting-up is very much like that of the Lancet; the type being small, and the matter arranged in columns. The page is however larger, and each number contains a sheet and a half. The annual price is only three dollars. It is divided into several departments: Original Papers, Reviews, Analyses of other Journals, Selections, Intelligence, &c. Judging from the numbers which have reached us, we should say that this Journal has hardly yet settled down into a determinate and fixed character, and suffers from the want of a recognized and responsible Editor. It seems to have been considerably improved latterly, as the recent numbers contain many valuable original communications and well-written reviews. We are, however, surprised to find one whole number (Sept. 1835,) composed of extracts from the English Journals; a facile mode, assuredly, of getting up a journal, and one which would save Editors a world of trouble and expense, if readers would be contented with it; but we should think our transatlantic brethren, honouring the land of their forefathers as they do, will hardly thank the Editor for such a wholesale transference of our pages as this. We have a suspicion that the publisher of the United States' Journal has too much to do with the editorial department of it; and that he has, moreover, chosen the emblem on his cover (*a lancet tranchant*) as congenial with his temper; at least, we infer as much from sundry bitter sayings on his wrapper in reference to his former editors. If we are right, we recommend him, if he wishes his journal to prosper, to put his Editors' names on his title-page, and to let them have their own way. But, as we shall occasionally voyage to "the United States" for goods to stock our third department withal, we must not take upon us to blame its governors, without further proof of their delinquency.

